# Bosma Arhinia Microphthalmia Syndrome (BAMS): First Report from Vietnam

**DOI:** 10.7759/cureus.35222

**Published:** 2023-02-20

**Authors:** Ngo Van Cong, Le Huu Nhat Minh, Le Huy Hoang, Uyen Do, Nguyen Thi My Dung

**Affiliations:** 1 Otolaryngology, Cho Ray Hospital, Ho Chi Minh City, VNM; 2 College of Medicine, Taipei Medical University, Taipei, TWN; 3 Research Center for Artificial Intelligence in Medicine, Taipei Medical University, Taipei, TWN; 4 Otolaryngology, University of Medicine and Pharmacy at Ho Chi Minh City, Ho Chi Minh City, VNM; 5 Nelda C. Stark School of Nursing, Texas Woman’s University, Houston, USA

**Keywords:** abnormalities, nose, choanal atresia, microphthalmia, arhinia, congenital arhinia syndrome, bosma arhinia microphthalmia syndrome

## Abstract

Bosma arhinia microphthalmia syndrome (BAMS) is a rare condition, with about 100 cases identified worldwide. It is characterized by nasal and ophthalmic abnormalities, as well as disturbances in puberty and sexual development. The cardinal sign is arhinia, though some cases have partial aplasia of the external nose. In addition, several reports have revealed abnormal brain structure, including changes to the olfactory bulbs. This case describes a 29-year-old female who has suffered from BAMS since birth. On presentation, she was noted to have congenital arhinia, bilateral microphthalmia, vision loss, mouth-breathing, an unclear speaking voice, a high arched or cleft palate, and a hypoplastic maxilla. Her paranasal sinuses were ossified and underdeveloped. This syndrome occurs rarely, both within Vietnam and worldwide. It is characterized by four major features: arrhinia, complete absence of the paranasal sinuses, eye defects, and absent sexual maturation. This case report describes the presentation of the disorder to improve otolaryngologists’ understanding of BAMS. Criteria for diagnosis consist of arhinia, midface hypoplasia (with a hypoplastic maxilla), hypogonadotropic hypogonadism, and normal intellectual abilities. Additional important findings are microphthalmia with or without coloboma, anosmia, maxillary hypoplasia, a high-arched palate, and absence of paranasal sinuses and olfactory bulbs.

## Introduction

Bosma arhinia microphthalmia syndrome (BAMS) was first described in 1981 [1]. It is a rare disease characterized by a combination of congenital arhinia (absence of the nose), microphthalmia, colobomas, and hypogonadism. Patients typically have normal brain structure and intellectual function [2]. There have been only around 100 cases of BAMS reported in medical literature [2]. Until now, its pathophysiology has not been clearly studied. However, these pathologies should always be differentiated from neoplasms, particularly squamous cell carcinoma (SCC) of the head and neck, which is the most common form of malignancy in this area [3]. The case described in this study is the first patient with BAMS to be identified in Vietnam, and potentially in the whole of Southeast Asia. We report this case to update and contribute to the clinical understanding of the manifestations of BAMS, particularly for otolaryngologists.

## Case presentation

**Figure 1:**
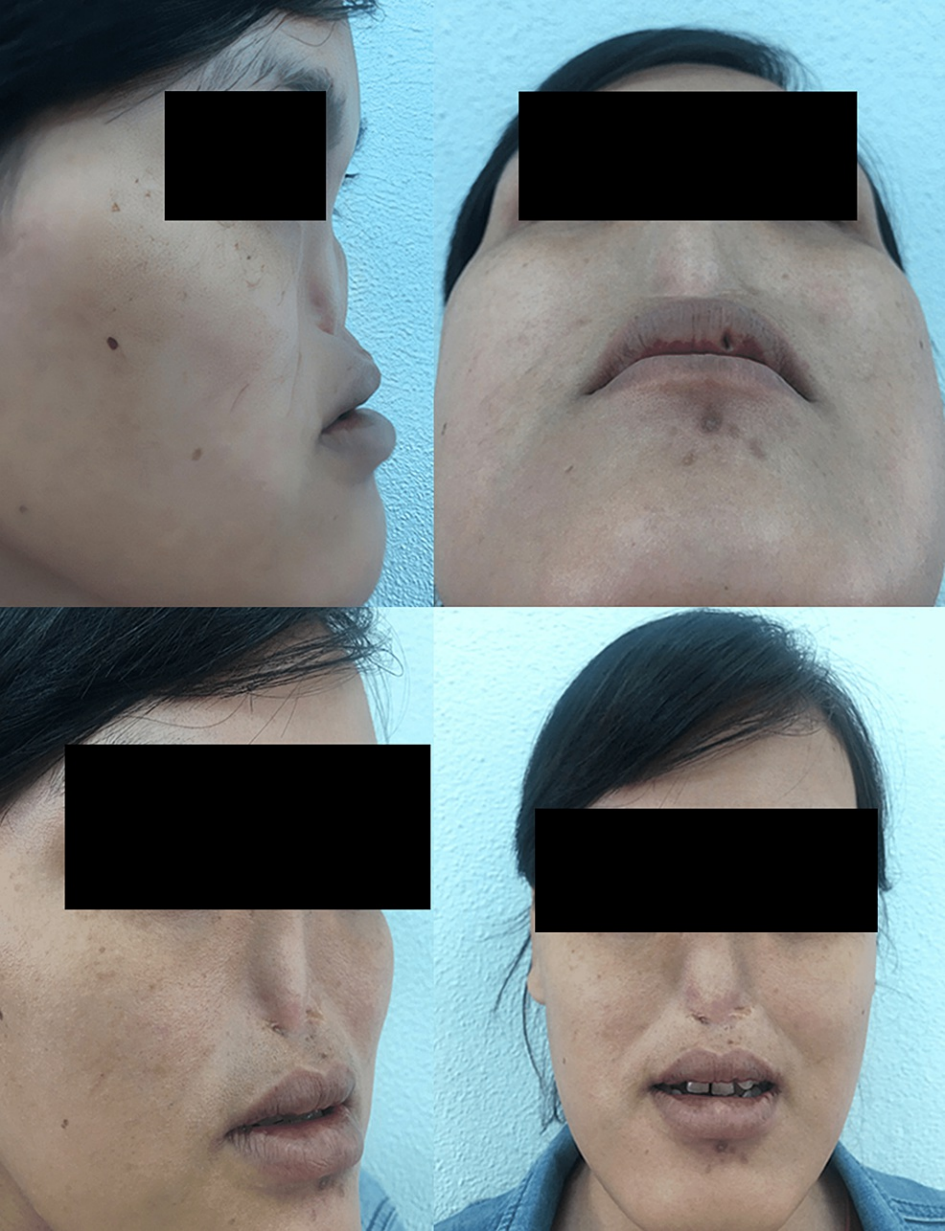
Arhinia and microphthalmia

A 29-year-old female presented to Cho Ray Hospital, Ho Chi Minh City, Vietnam, for arhinia, which was noted immediately at birth and remained underdeveloped. Past medical and family history were unremarkable. Examination revealed bilateral microphthalmia, 8/10 visual acuity in both eyes, and complete anosmia. Breathing was possible only through her mouth. She also had an unclear speaking voice, a short soft palate, and an undeveloped upper jaw (Figure 1). The symptoms had not deteriorated over the years.

**Figure 2:**
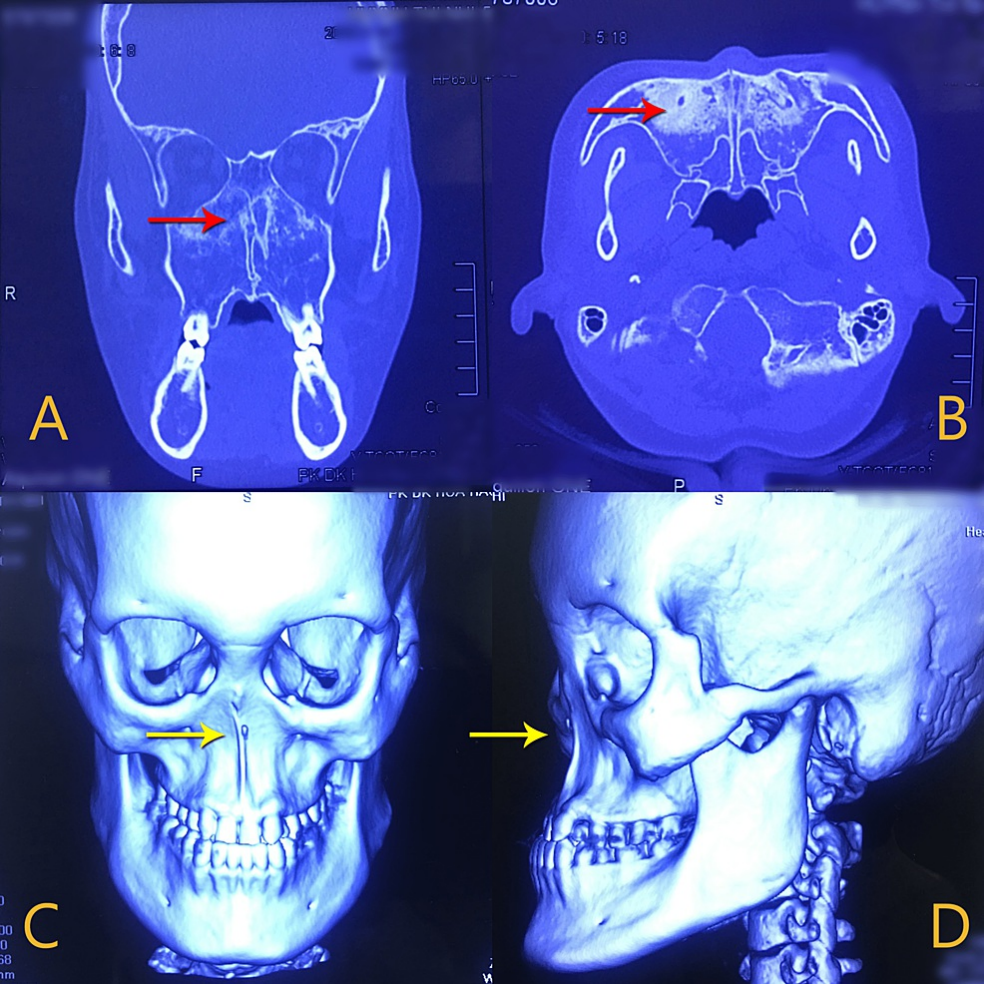
Sagittal view (a), axial view (b) and 3D view (c and d). CT images demonstrate atresia of the paranasal sinuses (red arrow) and flattened midface (yellow arrow).

Her external ears were normal, and hearing function was intact. A CT scan was performed to assess the olfactory sulci, olfactory bulbs, optic nerve, and adenohypophysis. Despite the fact that MRI can provide more information on imaging, the patient came to the hospital with the primary purpose of getting rhinoplasty and was not willing to undergo further tests such as MRI for other issues. Images showed the absence of paranasal sinuses with nearly confluent bone and nasal bones not developing (Figure 2). She had no previous laboratory testing or radiology to determine the presence of reproductive and other organs. She was very eager to undergo a rhinoplasty to raise her self-esteem when interacting with people. Notably, this marks the patient's initial consultation with Cho Ray Hospital and the first case of its kind in Vietnam. Our proposed treatment plan involved corrective nasal surgery to enhance aesthetic appeal, following which the patient can receive nasal reconstruction at the earliest feasible opportunity.

## Discussion

Clinical features

BAMS is a rare condition characterized by nasal and ophthalmic abnormalities, as well as disturbances in puberty and sexual development. The cardinal sign is arhinia, though some cases have partial aplasia of the external nose. In addition, several reports have revealed abnormal brain structure, including changes to the olfactory bulbs. Such clinical findings were supported by analyzing 14 patients from the several articles in literature [2,4-14]. In the study of Brasseur et al, all 14 patients were diagnosed with arhinia, with four out of the 14 exhibiting absence of olfactory bulbs. Additionally, six out of 14 patients had documented normal brain structures, except for the absence of olfactory bulb [2]. Patients with BAMS, therefore, typically suffer from hyposmia, anosmia, and ageusia, even though cognition and intellectual ability remain intact.

Ophthalmic findings in BAMS include bilateral microphthalmia or even anophthalmia, causing decreased or complete loss of visual acuity in most patients. These abnormalities are due to defects in several structures of the ocular lens or crystalline lens [2].

In previous studies, the patient's quality of life can be seriously impacted by physical damage resulting from tumors or genetic structural abnormalities in the nasal region [15,16]. For instance, BAMS in a 40-year-old male can induce social dissatisfaction, including feelings of unhappiness, abandonment, loneliness, and emotional distress due to physical differences experienced during childhood. Additionally, craniofacial defects may also occur in Bosma patients, which include a high-arched palate, absent nasal or paranasal sinuses, choanal atresia, nasolacrimal duct stenosis, and a small maxilla. These defects cause patients to have difficulty with breathing and a negative impact on their quality of life, particularly as children. On occasion, some cases can have external ear abnormalities [16].

According to Brasseur et al, several BAMS cases present with hypoplasia of the reproductive system due to decreased gonadotropin levels [2]. This results in hypogonadism, directly affecting genital development. If left untreated, low levels of these hormones may lead to delayed puberty (even in males), undeveloped genital organs, and cryptorchidism. Decreased gonadotropin levels also result in low bone density, leading to osteoporosis and fragility fractures when exposed to loads.

Studies by Mart & McNerny, 2013 [17] and Paterick et al., 2013 [18] found that echocardiography of patients with BAMS may reveal effacement of the sinotubular ridge, which requires frequent follow-up. This condition is well recognized in connective tissue diseases such as Marfan syndrome, and in patients with bicuspid aortic valves. Thiele et al. (1996) [19] reported patent ductus arteriosus in a patient with arhinia, microphthalmia, a high arched palate, and absence of paranasal sinuses, but with normal cognition. The ductus arterioles is a blood vessel connecting the aorta and the pulmonary artery. The finding of patent ductus arterioles in BAMS is significant as it is suggestive of a multi-organ defect condition. 

Incidence

BAMS is a very rare disease, and its incidence is unknown. The incidence between males and females is unknown. Less than 100 cases have been described in historic literature, and it has not been reported at all in some populations [2]. This patient does not exhibit any laryngeal deformities or abnormalities and is able to breathe normally through their mouth, meaning there is no difference in the rate of tracheostomy compared to that of healthy individuals.

Etiology

Most BAMS cases are caused by a mutation in the SMCHD1 gene. SMCHD1 encodes a uncharacterized protein that has a silencing effect, and is involved in the development of the eyes, nose, and other craniofacial structures [20].

Researchers have not yet confirmed the mechanisms by which the SMCHD1 gene mutation affects the functions of its protein product and induces the abnormalities found in BAMS. The mutation may lead to silencing of its own gene expression, resulting in the arhinia, microphthalmia, and craniofacial abnormalities of BAMS. Nasal development can be influenced by the release of gonadotropin-releasing hormone (GnRH). Subsequently, gonadotropin, which controls the production of reproductive hormones, is also affected. The decreased levels of gonadotropin may explain the hypoplasia of these patients’ genital organs [2].

It should be noted, however, that some patients with SMCHD1 mutations have presented with arrhinia, but not the other characteristics associated with BAMS, or with only mild nasal defects. Researchers, therefore, suspect that other genetic factors may play a role in producing these symptom. However, this area has not been studied in depth.

Management

BAMS may not be too difficult to diagnose because of the arhinia feature. Therefore, its early recognition and diagnosis is critical. To date, disease management remains unspecific and mainly involves full investigation of nasal, ophthalmic, ocular, cardiac, and reproductive defects, followed by appropriate surgical intervention. Particularly in female patients, genital organs should be examined and treated for any abnormality to improve reproductive capacity. Patients should also be provided with necessary vitamins to prevent osteoporosis. Careful follow-up and management of associated abnormalities is essential, depending on the severity of their presentation. Structural defects may be reconstructed using plastic surgery once patients reach adulthood [9].

## Conclusions

BAMS is a rare disease, with very few cases identified worldwide. It has many different types of damage to various organs. Criteria for diagnosis consists of arhinia, midface hypoplasia (with a hypoplastic maxilla), hypogonadotropic hypogonadism, and normal intellectual abilities. Additional important findings are microphthalmia with or without coloboma, anosmia, maxillary hypoplasia, a high-arched palate, and absence of paranasal sinuses and olfactory bulbs. Therefore, we introduced a typical case to enable clinicians to recognize and approach it comprehensively without being confused in diagnosis and approach of the damage to the patient.
